# Antifungal Enantiomeric Styrylpyrones from *Sanrafaelia ruffonammari* and *Ophrypetalum odoratum*

**DOI:** 10.1007/s13659-014-0014-6

**Published:** 2014-04-15

**Authors:** Hamisi M. Malebo, Charles Kihampa, Clarence A. Mgina, Fortunatus Sung’hwa, Reiner Waibel, Stephan A. Jonker, Mayunga H. H. Nkunya

**Affiliations:** 1 Department of Traditional Medicine Research, National Institute for Medical Research, P.O. Box 9653, Dar es Salaam, Tanzania; 2 Department of Environmental Science and Management, Ardhi University, P.O. Box 35176, Dar es Salaam, Tanzania; 3 Department of Chemistry, University of Dar es Salaam, P.O. Box 35061, Dar es Salaam, Tanzania; 4 Department of Pharmaceutical Chemistry, Institute of Pharmacy, University of Erlangen, Schuhstrasse 19, 91052 Erlangen, Germany

**Keywords:** *Sanrafaelia ruffonammari* Verd, *Ophrypetalum odoratum* Diels, Styrylpyrones, Antifungal

## Abstract

Phytochemical investigation of *Sanrafaelia ruffonammari* Verd and *Ophrypetalum odoratum* Diels that belongs to the rare genera confined to East African coastal forests led to the isolation of enantiomeric styrylpyrone dimer, (±)-5-methoxy-7-phenyl-[4-methoxy-2-pyronyl]-1-(*E*)-styryl-2-oxabicyclo-[4.2.0]-octa-4-en-3-one (**1**) alongside (+)-6-styryl-7,8-epoxy-4-methoxypyran-2-one (**2**) and the enantiomeric (+)- (**3**) and (−)-6-styryl-7,8-dihydroxy-4-methoxypyran-2-ones (**4**). Their structures were established by means of spectroscopic methods. In this paper we reveal for the first time the occurrence of styrylpyrones in East African biodiversity. (+)-6-Styryl-7,8-epoxy-4-methoxypyran-2-one (**2**) and the dihydroxystyrylpyrone enantiomer (**3**) showed in vitro antifungal activity against *Candida albicans* at a concentration of 24.4 and 26.2 µM with zones of inhibition of 17 and 9 mm, respectively. Compound **2** exhibited strong activity in the brine shrimp test with LC_50_ = 1.7 µg/mL. Their high cytotoxic and antifungal activities render them candidates for further scientific attention for drug development programs against cancer and microbial infections.

## Introduction

*Sanrafaelia ruffonammari* Verd and *Ophrypetalum odoratum* Diels belong to the rare genera that are confined to East African coastal forests, of which *S.* *ruffonammari* was only recently described by Verdcourt, 1999 [1–3]. The plant species are not reported to be used in traditional medicine. In our recent investigation, the ethanol extracts from the root bark of *S.* *ruffonammari* and *O.* *odoratum* exhibited cytotoxicity in the brine shrimp test (IC_50_ = 79 and 1.29 μg/mL, respectively). These results and the fact that both plant species are rare and hence threatened with extinction prompted us to investigate them for their chemical constituents, as part of our on-going phytochemical studies of Annonaceae species growing in Tanzania. From *O.* *odoratum* stem and root barks, and leaves we have isolated an enantiomeric styrylpyrone dimer, (±)-5-methoxy-7-phenyl-[4-methoxy-2-pyronyl]-1-(*E*)-styryl-2-oxabicyclo-[4.2.0]-octa-4-en-3-one (**1**), (+)-6-styryl-7,8-epoxy-4-methoxypyran-2-one (**2**) and the enantiomeric (+)- (**3**) and (−)-6-styryl-7,8-dihydroxy-4-methoxypyran-2-ones (**4**), while the root bark of *S.* *ruffonammari* yielded (+)-6-styryl-7,8-epoxy-4-methoxypyran-2-one (**2**) and (−)-6-styryl-7,8-dihydroxy-4-methoxypyran-2-one (**3**). 
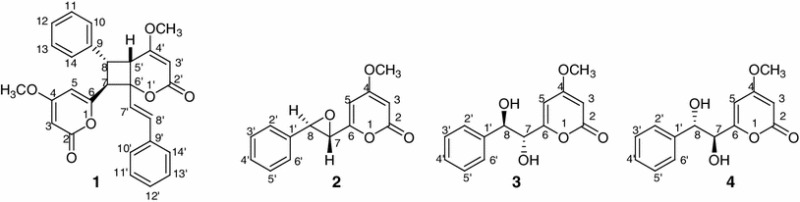


## Results and Discussion

The EtOH extracts of the air-dried and powdered root bark of *S.* *ruffonammari* and stem and root barks, and leaves of *O.* *odoratum* were separately fractionated by vacuum liquid chromatography (VLC) over silica gel and repeated column chromatography on silica gel and Sephadex LH-20 column to afford the enantiomeric styrylpyrone dimer, (±)-5-methoxy-7-phenyl-[4-methoxy-2-pyronyl]-1-(*E*)-styryl-2-oxabicyclo-[4.2.0]-octa-4-en-3-one (**1**), (+)-6-styryl-7,8-epoxy-4-methoxypyran-2-one (**2**) and the enantiomeric (+)- (**3**) and (−)-6-styryl-7,8-dihydroxy-4-methoxypyran-2-ones (**4**) from the stem and root barks, and leaves of *O.* *odoratum* while the root bark of *S.* *ruffonammari* yielded (+)-6-styryl-7,8-epoxy-4-methoxypyran-2-one (**2**) and (−)-6-styryl-7,8-dihydroxy-4-methoxypyran-2-one (**3**). The structures of styrylpyrones were elucidated by extensive spectroscopic methods and comparison with literature data [4–8].

The ethanolic crude extract of *O.* *odoratum* root bark exhibited potent bioactivity in the brine shrimp lethality test with an LC_50_ value of 1.29 µg/mL. The ethanol extract of the root bark of *S.* *ruffonammari* showed a very mild lethality to shrimp larvae with an LC_50_ value of 79 µg/mL. Styrylpyrone **2** exhibited a moderate antifungal activity against *Candida albicans* at a concentration of 24.4 µM with zone of inhibition of 17 mm. In the brine shrimp test, compound **2** exhibited strong bioactivity with IC_50_ value of 1.7 µg/mL. It was however inactive against *Trypanosoma brucei rhodesiense* with minimum inhibition concentration of 56 µg/ml. Styrylpyrone **3** exhibited mild activity against *C.* *albicans* at a concentration of 26.2 µM, with zone of inhibition of 9 mm in the plate diffusion test.

Styrylpyrones have been reported to occur in plant species of the families Annonaceae and Zingiberaceae [7, 8] and more recently, in the *Phellinus* and *Inonotus* genera of medicinal mushrooms [9]. Styrylpyrones isolated from medicinal fungi, including *P.* *linteus*, *P.* *igniarius*, *P.* *ribis*, *I.* *obliquus* and *I.* *xeranticus* exhibited various biological activities, including anti-oxidative, anti-inflammatory, cytotoxic, anti-platelet aggregation, anti-diabetic, anti-dementia and anti-viral effects [9]. There is a massive body of evidence indicating that styrylpyrones display in vitro cytotoxic effect especially by inducing apoptosis in different cancer cell lines including breast, colon, kidney and pancreatic carcinoma cells [9–12]. The mode of action of styrylpyrones as anticancer agents mainly targets the mitochondria [10].

In recent years, styrylpyrones have received more scientific attention due to their remarkable cytotoxic and antitumor properties against various human tumor cell lines such as lung carcinoma cells A-549, promyelocytic leukemia cells HL-60 and stomach cancer cells SGC-7901 [10–12]. Studies on the mechanism of action of a styrylpyrone goniothalamin indicated that it induces apoptosis in MCF-7 breast cancer and HL-60 human cancer cells [12–15]. Styrylpyrones have been identified as an interesting group of bioactive compounds with unique carbon skeleton which might be an attractive molecular scaffold for cytotoxic drug design and synthesis hence promising pharmacological applications against several mammalian cancer cell lines [13–16].

The existence of styrylpyrones in *S.* *ruffonammari* and *O.* *odoratum* occurring in Tanzania and that, this class of compounds exhibit anticancer, antifungal, antibacterial and antiviral activities provides strong evidences for further consistent and systematic research on these genera as it might lead to the discovery of antineoplastic and antimicrobial agents. Such investigation might also provide a pool of chemical compounds critical for future biological target studies. If enough botanical, phytochemical and pharmacological work is dedicated to these rare tropical genera of flowering plants, a couple of new drugs for the treatment of tumors, fungal, bacterial and even viral infections can be developed in the relatively close future.

## Experimental Section

### General Experimental Procedures

Optical rotations were measured on Jasco-P-1020 polarimeter. Infrared (IR) spectra, taken in chloroform solutions were recorded on a Shimadzu Model IR-435 spectrophotometer with absorptions given in wave numbers (cm^−1^). ^1^H NMR spectra were recorded on either a Bruker AM 360 instrument operating at 360 MHz with CDCl_3_ was used as solvent at the Institute of Pharmacy, University of Erlangen in Germany. Column chromatography was carried out with silica gel (200–300 mesh) and Sephadex LH-20 (Amersham Biosciences, Sweden). Fractions were monitored by Thin layer chromatography (TLC). Visualization of TLC spots was done under UV light at 254 or 366 nm and by spraying with an anisaldehyde reagent. Detection was done under UV light at 254 or 366 nm. Vacuum liquid chromatography (VLC) was carried out using normal phase silica gel [of particle size 400 Mesh ASTM (Merck)] and gradient elution was applied. The vacuum was generated from a membrane pump.

### Plant Materials

The root bark of *S.* *ruffonammari* was collected from Kwamtiri forest in the East Usambara Mountains, Tanzania. Leaves, stem and root barks of *O.* *odoratum* were collected from Kiloka Pass in Morogoro and from Pugu Forest Reserve, Tanzania. Mr. L.B. Mwasumbi, a plant taxonomist at the Herbarium of the Department of Botany, University of Dar es Salaam identified all plant materials. Voucher specimens are deposited at the above Herbarium.

### Extraction and Isolation

The air-dried powdered plant materials were soaked consecutively in petrol, chloroform and ethanol for 2 × 48 h. The extract from the root bark of *S.* *ruffonammari* and leaves of *O.* *odoratum* were separately fractionated by vacuum liquid chromatography (VLC) over silica gel and repeated column chromatography on silica gel and Sephadex LH-20 yielded the enantiomeric styrylpyrone dimer, (±)-5-methoxy-7-phenyl-[4-methoxy-2-pyronyl]-1-(*E*)-styryl-2-oxabicyclo-[4.2.0]-octa-4-en-3-one (**1**), (+)-6-styryl-7,8-epoxy-4-methoxypyran-2-one (**2**) and the enantiomeric (+)- (**3**) and (−)-6-styryl-7,8-dihydroxy-4-methoxypyran-2-ones (**4**) from the stem and root barks, and leaves of *O.* *odoratum* while the root bark of *S.* *ruffonammari* yielded (+)-6-styryl-7,8-epoxy-4-methoxypyran-2-one (**2**) and (−)-6-styryl-7,8-dihydroxy-4-methoxypyran-2-one (**3**).

### Brine Shrimp Test (BST)

The brine shrimp test to evaluate the cytoxicity of crude extracts and pure compounds was carried out using brine shrimps (*Artemia salina*) larvae as test organisms was carried out using the method on Meyer et al. [17].

### Antifungal Tests

The antifungal assay to evaluate the ability of the pure compounds to inhibit growth of *C.* *albicans* in a culture media was carried out using the standard plate diffusion method. The medium was prepared as follows: 32.50 g of Sabouraud Dextrose Agar (SDA) was mixed with 500 mL of sterile distilled water. The mixture was sterilized by autoclaving at 120 °C for 15 min under 1 bar pressure. Under aseptic conditions in the laminar flow hood, the medium was dispensed into 150 mm pre-sterilized petri dishes to yield a uniform depth of 4 mm. They were then covered and allowed to cool and hardened at room temperature. The hardened medium was inverted and then incubated at 37 °C for the sterility assurance test. The microbial nutrient broth (2 g) was mixed with 250 mL of sterile distilled water. The mixture was sterilized by autoclaving at 120 °C for 15 min under 1 bar pressure. The nutrient broth was cooled, and an innoculum from a pure subculture of a *C.* *albicans* colon was innoculated into the broth and then diluted threefold, then introduced into the culture medium. Four circular wells were made in each culture medium and 10 µL containing 100 µg/mL of pure compounds dissolved in dimethyl sulfoxide (DMSO) was dispensed into each of the three wells in the medium, the fourth one being dispensed with 10 µL of DMSO, as a control. After the compounds had diffused into the medium, the culture medium was inverted and incubated at 37 °C for 24 h. The absence of a clear circular region around the disc loaded with a measured volume of test compound was used as an indicator of growth. The inhibition zone was determined by measuring the diameter in millimetres of the circular region around each well.

### In vitro Anti-trypanosomal Assay

The in vitro anti-trypanosomal activity was evaluated against *Trypanosoma brucei rhodesiense* strain, using the cultivation method of Baltz et al. [18].

### (±)-5-Methoxy-7-phenyl-[4-methoxy-2-pyronyl]-1-(*E*)-styryl-2-oxabicyclo-[4.2.0]-octa-4-en-3-one (**1**)

Yield: 39 mg; m.p. 191 °C. Anisaldehyde: Pink. [α]_D_ = 0°. UV, ν_max_ 210 and 290 nm. IR, ν_max_ 1708, 1642, 1565, 1452, 1390, 1237, 1211, 986 and 963 cm^−1^. ^1^H NMR: δ 3.27 (s, 3H, OCH_3_), 3.60 (d, 1H, J = 9.70 Hz, H-5′), 3.71 (s, 3H, OCH_3_), 4.16 (d, 1H, J = 10.98 Hz, H-7), 4.36 (dd, 1H, J = 10.98, 9.70 Hz, H-8), 5.29 (s, 1H, H-3′), 5.34 (d, 1H, J = 2.16 Hz, H-3), 5.91 (d, 1H, J = 2.16 Hz, H-5), 6.62 (d, 1H, J = 15.86 Hz, H-7′), 6.95 (d, 1H, J = 15.86 Hz, H-8′) and 7.43–7.23 (m, 10H, 2 × C_6_H_5_). ^13^C NMR, δ 170.4 (C-4), 169.8 (C-4′), 64.5 (C-2), 163.8 (C-2′), 158.6 (C-6), 135.8 (C-9 and C-9′), 131.4 (C-8′), 128.7 (C-11 128.4 (C11′ and C-13′), and C-13), 128.2 (C-12), 127.8 (C-12′), 127.5 (C-10 and C-14), 126.8 (C-10′ and C-14′), 124 (C-7′), 91.7 (C-3), 88.6 (C-3′), 79.3 (C-6′), 55.8 (C-15), 55.3 (C-15′), 54.4 (C-8), 45.7 (C-7) and 39.1 (C-5′). MS, *m*/*z*, (% rel. int.), 456 ([M]^+^), 228 (100, [C_14_H_12_O_3_]^+^), 200 (55, [C_13_H_12_O_2_]^+^), 185 (25), 157 (45, [C_11_H_9_O]^+^), 129 (25), 103 (25, [C_8_H_6_]^+^), 77 (18, [C_6_H_5_]^+^).

### (+)-6-Styryl-7,8-epoxy-4-methoxypyran-2-one (**2**)

Yield: 88 mg from root bark, 60 mg from stem bark; m.p. 110 °C. [α]_D_ = + 136.2° (0.6, CHCl_3_).Anisaldehyde: Pink. IR ν_max_ 3400, 3083, 2939, 1717, 1643 and 1567 cm^−1^. MS, *m*/*z* (% rel. int.) 245 ([M]^+^, 9), 244 (30), 228 (17), 215 (17), 188 (18), 187 (60), 157 (12), 155 (20), 138 (100), 125 (40), 110 (60), 112 (22), 95 (65), 80 (40), 77 (47), 69 (81), 59 (20) and 52 (30). UV, ν_max_ 211, 224, 241 and 287 nm. ^1^H NMR and ^13^C NMR see Tables 1 and 2.

**Table 1:** ^1^H NMR spectral data of compounds **2**–**4**

H	**2**		**3**		**4**	
	*δ*	*J* (Hz)	*δ*	*J* (Hz)	*δ*	*J* (Hz)
3	5.50	*d*, 2.5	5.37	*d*, 2.7	5.40	*d*, 2.7
5	6.10	*d*, 2.5	5.90	*dd*, 2.7, 1.8	5.90	*dd*, 2.5, 1.8
7	4.15	*d*, 2.0	5.12	*d*, 3.5	5.15	*dd*, 4.9, 5.0
8	3.62	*d*, 2.0	4.67	*d*, 3.5	4.65	*dd*, 5.0, 5.0
OCH_3_	3.80	*s*	3.73	*s*	3.74	*s*
7-OH	–	*s*	3.31	*s*	2.80	*d*, 4.9
8-OH	–	*s*	3.31	*s*	2.85	*d*, 5.0
2′–6′	7.25	*m*	7.25	*m*	7.35	*m*

**Table 2:** ^13^C NMR spectral data of compounds **2**–**4**

C	*δ*		
	**2**	**3**	**4**
2	170.61	171.07	171.08
3	89.27	88.40	88.39
4	163.40	164.17	164.19
5	100.26	100.76	100.77
6	159.27	162.45	162.50
7	60.54	74.94	74.93
8	58.06	74.16	74.17
1′	135.03	138.56	138.56
2′, 6′	128.65	128.42	128.42
3′, 4′	125.86	126.69	126.69
5′	128.92	128.42	128.42
OCH_3_	56.01	55.92	55.93

### (−)-6-Styryl-7,8-dihydroxy-4-methoxypyran-2-one (**3**)

Overall yield 317 mg (0.02 % based on dry leaves) m.p. 137 °C. [α]_D_ = + 126.9° (0.6, CHCl_3_). UV, ν_max_ 210 and 290 nm, IR, ν_max_ 3573, 3353, 1708, 1664, 1567, 1452, 1409, 1245, 1031, 811 and 699 cm^−1^. ^1^H MS, *m*/*z* (% re int) 262 (M^+^), 156 (100, C_7_H_8_O_4_^+^), 127 (30), 107 (10, C_7_H_7_O^+^), 79 (25), 69 (18), 59 (10) and 39 (10). ^1^H NMR and ^13^C NMR data see Tables 1 and 2.

### (+)-6-Styryl-7,8-dihydroxy-4-methoxypyran-2-one (**4**)

Yield: 317 mg from leaves, m.p. 137 °C. Anisaldehyde: Pink. [α]_D_ = + 126.9° (0.6, CHCl_3_). UV, λ_max_ 210, 226 and 290 nm, IR, υ_max_ 3573, 3353, 1708, 1664, 1567, 1452, 1409, 1245, 1031, 811 and 699 cm^−1^. MS, *m*/*z* (% rel. int.) 262 ([M]^+^, 9), 156 (100, [C_7_H_8_O_4_]^+^), 127 (30), 107 (10, [C_7_H_7_O]^+^), 79 (25), and 69 (18). ^1^H NMR and ^13^C NMR see Tables 1 and 2.
